# A model with multiple intracranial aneurysms: possible hemodynamic mechanisms of aneurysmal initiation, rupture and recurrence

**DOI:** 10.1186/s41016-024-00364-5

**Published:** 2024-05-06

**Authors:** Wenqiang Li, Chao Wang, Yanmin Wang, Yapeng Zhao, Xinjian Yang, Xianzhi Liu, Jian Liu

**Affiliations:** 1 Department of Interventional Neuroradiology and Neurosurgery, Beijing Neurosurgical Institute and Beijing Tiantan Hospital, Beijing, China; 2 Department of Neurosurgery, The First Affiliated Hospital of Zhengzhou University, Zhengzhou, China

**Keywords:** Multiple intracranial aneurysms, Hemodynamic, Aneurysm initiation and growth, Rupture, Recurrence

## Abstract

**Background:**

Hemodynamic factors play an important role in aneurysm initiation, growth, rupture, and recurrence, while the mechanism of the hemodynamic characteristics is still controversial. A unique model of multiple aneurysms (initiation, growth, rupture, and recurrence) is helpful to avoids the confounders and further explore the possible hemodynamic mechanisms of aneurysm in different states.

**Methods:**

We present a model with multiple aneurysms, and including the states of initiation, growth, rupture, and recurrence, discuss the proposed mechanisms, and describe computational fluid dynamic model that was used to evaluate the likely hemodynamic effect of different states of the aneurysms.

**Results:**

The hemodynamic analysis suggests that high flow impingement and high WSS distribution at normal parent artery was found before aneurysmal initiation. The WSS distribution and flow velocity were decreased in the new sac after aneurysmal growth. Low WSS was the risk hemodynamic factor for aneurysmal rupture. High flow concentration region on the neck plane after coil embolization still marked in recanalized aneurysm.

**Conclusions:**

Associations have been identified between high flow impingement and aneurysm recanalization, while low WSS is linked to the rupture of aneurysms. High flow concentration and high WSS distribution at normal artery associated with aneurysm initiation and growth, while after growth, the high-risk hemodynamics of aneurysm rupture was occurred, which is low WSS at aneurysm dome.

## Background

Hemodynamics have been proven to contribute significantly to intracranial aneurysm initiation, growth, rupture, and recurrence [1–4]. Studying a single patient with multiple aneurysms exhibiting aneurysmal initiation, rupture, and recurrence over the course of their treatment could provide a unique hemodynamic model that avoids many confounders, such as effects of blood pressure and the vascular conditions. However, such cases are extremely unique. To our knowledge, there have been no reported hemodynamic analyses of such aneurysms to date.

In the current study, we present a case with multiple aneurysms, including four intracranial aneurysms: (1) a ruptured anterior communicating artery (AcomA) aneurysm, which was initially embolized successfully and underwent recanalization at an 18-month follow-up; (2) a newly-initiated basilar tip aneurysm, which had grown and underwent rupture at an 18-month follow-up; (3–4) bilateral posterior communicating artery (PcomA) aneurysms located on the left and right sides, which were successfully embolized and clipped, respectively, and were stable on an 18-month follow-up angiograph. Employing computational fluid dynamics (CFD), we performed simulations before and after the embolization, and before and after the initiation. We aimed to decode the hemodynamic signatures pertinent to the aneurysms' progressive states.

## Methods

### Model report

**Fig. 1:**
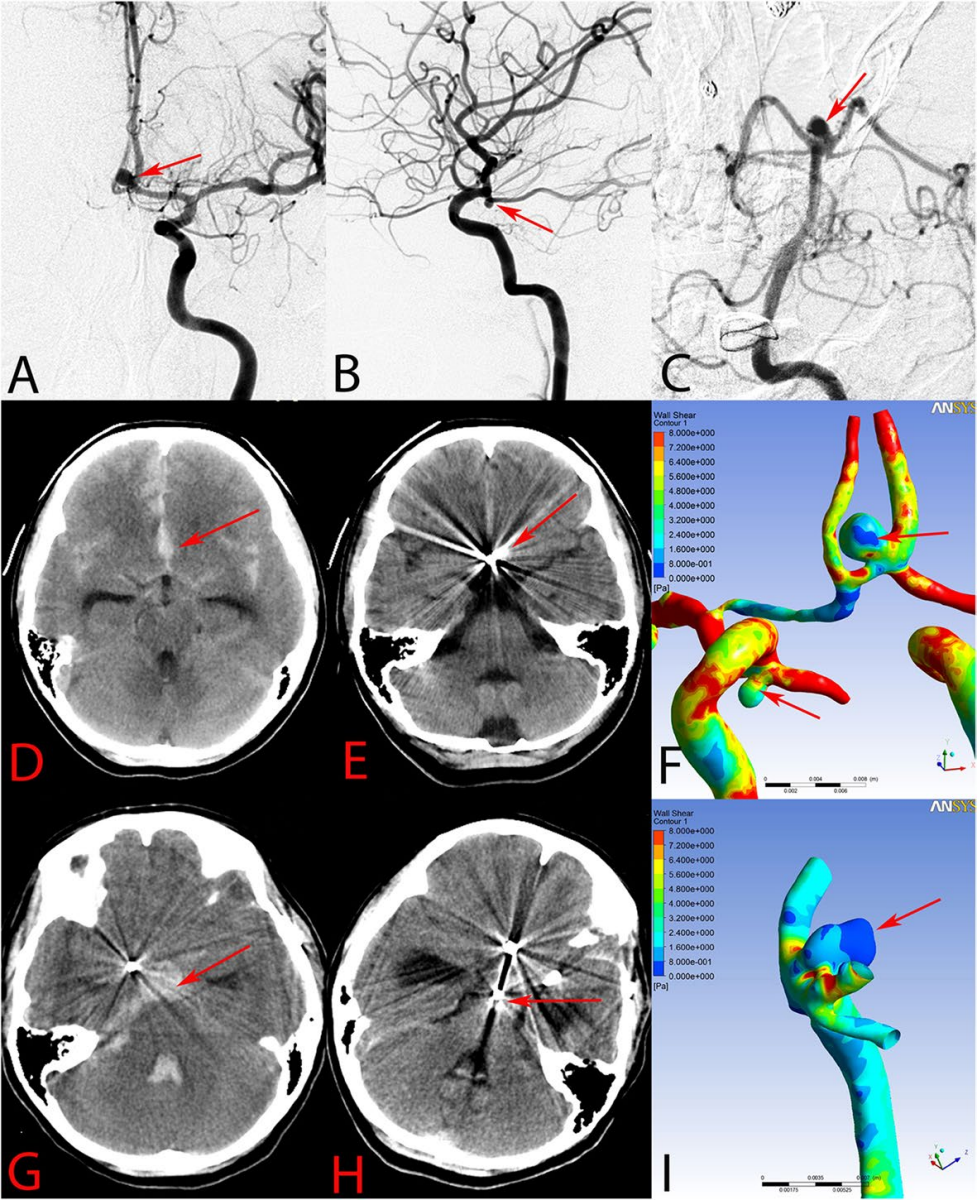
Hemodynamic characteristics of the aneurysmal rupture. Three aneurysms were coiled in this case (arrows in **A**, **B**, and **C**). Twice subarachnoid hemorrhage (SAH) was occurred in the patient. The first SAH was caused by the anterior communicating artery aneurysm (**D**, **E**), while for the second SAH, the basilar artery tip aneurysm was considered to be the ruptured aneurysm (**G**, **H**). The hemodynamic characteristics of the ruptured aneurysm dome showed a lower wall shear stress magnitude than the unruptured aneurysm (arrows in **F** and **I**)

**Fig. 2:**
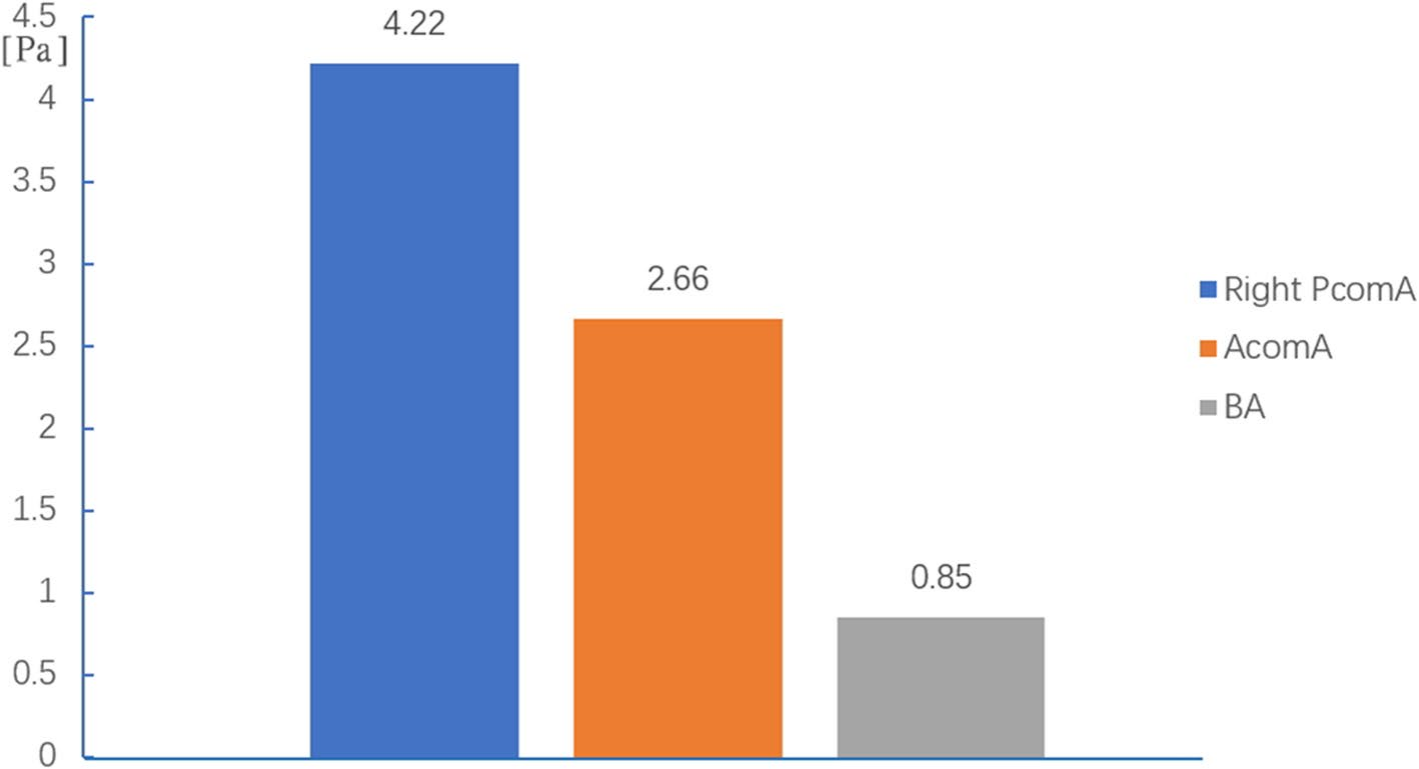
The values of wall shear stress magnitudes of the three aneurysms. (posterior communicating artery (PcomA); anterior communicating artery (AcomA); Basilar artery (BA))

A 30-year-old female presented with subarachnoid hemorrhage (SAH) occurring within a day. Her medical background included hypertension and hyperlipidemia, with no reported cases of diabetes or ischemic cerebrovascular conditions. The patient had overweight with BMI=27.34 kg/m^2^. She was graded 4 on the Hunt-Hess scale, and 5 on the Modified Rankin scale (mRS). Standard digital subtraction angiography (DSA) and three-dimensional (3D) rotational angiography were performed. Three intracranial aneurysms were found: (1) a 3.7 × 3.8 mm left AcomA aneurysm; (2) a 1.7 × 2.2 mm PcomA segment aneurysm; and a 2.0 × 2.4 mm right PcomA segment aneurysm. According to the head computed tomography (CT) scan, the AcomA aneurysm was identified as ruptured (Fig 1D, E). It was embolized with coils alone, and the right PcomA aneurysm was also treated endovascularly with coiling alone. At the end of the procedure, the lesions were totally embolized without procedure complication (Fig 2B, F). A week later, the left PcomA aneurysm was clipped without procedure complication.

**Fig. 3:**
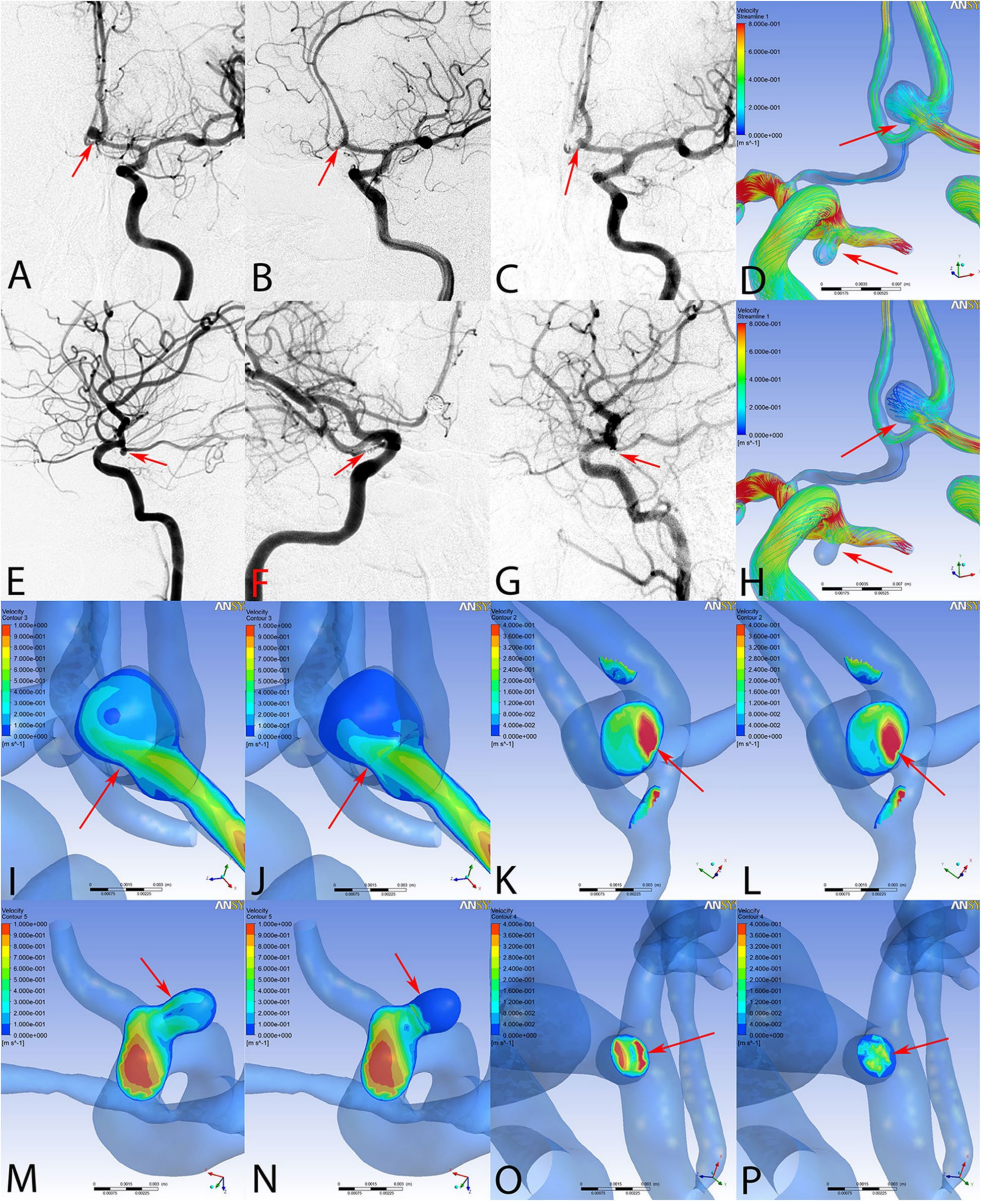
Hemodynamic characteristics of the aneurysmal recanalization. In pre-procedure, immediate post-procedure, and 18-month follow-up angiographic images, the anterior communicating artery (Acom) showed recanalization (arrows in **A**–**C**), while the left posterior communicating artery (Pcom) was still completely occluded (arrows in **E**–**G**). The hemodynamic alterations are shown comparing the pre-procedure (**D**, **I**, **K**, **M**, and **O**) and post-procedure (**H**, **J**, **L**, **N**, and **P**) images. Compared with pre-procedure result, the Streamlines results show that the flow of the Acom aneurysm was still evident (upper arrows in **D** and **H**), while the flow of the Pcom aneurysm was well-modified (lower arrows in **D** and **H**). The velocity profiles at both the neck and the sac of the aneurysm are additionally illustrated. The Acom aneurysm displayed a concentration of maximum blood flow at the neck area, persisting near the inflow region where recanalization later emerged, as observed in follow-up images. In contrast, the Pcom aneurysm presented a considerable decrease in velocity, lacking any areas of increased or focal velocity on the sectional plane of the neck.(arrows in **M**–**P**)

No ischemic and hemorrhagic complications occurred after the operations. The patient was discharged three weeks later with an mRS score of 0. Follow-up angiography at six months was recommended. However, the patient did not comply. At 18 months after the treatment, the patient suffered from a intense headache and suffered SAH again, as verified by a CT scan (Fig 1G, H). On this occasion, she was graded 5 on the Hunt-Hess scale and 5 on the Modified Rankin scale. Standard DSA and 3D rotational angiography were performed. A new 2.5 × 4.4 mm basilar tip aneurysm with a daughter bleb was found, showing a rupture lesion (Fig 1C). The aneurysm was subtotally embolized with stent-assisted coiling. In addition, the angiograph results showed that the PcomA aneurysms were stable and were totally occluded (Fig 3G). However, the AcomA aneurysm was recanalized with a residual neck and did not treated simultaneously (Fig 3C). After the procedure, the patient remained in a coma. Unfortunately, 24 d later, after the second SAH, the patient suffered sudden cardiac arrest and died.

### Computational modeling, hemodynamic simulations, and hemodynamic analysis

The patient's aneurysm volume was reconstructed from three-dimensional digital subtraction angiography (3D DSA) data. Using the Geomagic Studio 12.0 software suite, we rendered the 3D surface geometry, which was then segmented and refined for smoothness. To approximate the effect of the embolization coils within the dome of the aneurysm, we applied the porous media approach [5]. We then imported this refined aneurysmal geometry into ICEM CFD software (ANSYS) to generate a mesh of finite volume tetrahedral elements, supplemented by prism layers to aid in computational fluid dynamics (CFD) simulation accuracy. Mesh complexity varied, with the largest elements capped at 0.2 mm and total element counts ranging from 2.8 to 5.2 million. To model post-treatment conditions, we merged geometries of the aneurysm dome pre-embolization with those of the coiled parent vessel post-treatment. Additionally, the aneurysmal dome containing coils was represented as a porous medium, as previously reported by us [6]. The parameter setting in coil modeling was determined based on the clinical utilization of coils [7]. The hemodynamics were simulated using CFX 14.0 software (ANSYS) following meshing. The calculation was guided by fundamental equations rooted in the Navier–Stokes formulation, assuming homogeneous, laminar, and incompressible blood flow. Blood flow was considered to adhere to the characteristics of a Newtonian fluid, while the blood vessel wall was presumed to be rigid, adhering to no-slip boundary conditions. The density of the blood was set at ρ = 1,060 kg/m^3^, whereas its dynamic viscosity was designated as μ = 0.004 Pa·s. Using transcranial Doppler imaging, a representative pulsatile velocity profile over a period was acquired and subsequently established as the inflow boundary condition. A representative pulsatile velocity profile over a period was obtained using transcranial Doppler imaging and was then utilized to define the inflow boundary condition. In our study, the outlet pressure conditions at the arterial outlets were set to p = 0 Pa. Additionally, the flow waveforms were adjusted to attain an average inlet WSS of 15 dyne/cm under pulsatile conditions. To reduce initial transients and confirm the numeric stability, we computed 3 complete cardiac cycles, each cardiac cycle was divided into 800 time steps, with a duration of 0.001 seconds per step. For the final analyses, the outcomes obtained from the third cardiac cycle were gathered as the output.

To characterize the flow conditions within the aneurysm for each stage, we utilized the WSS and flow velocity at peak systole. Flow impingement and inflow concentration were defined as Cebral et al. [8] reported. The flow impingement zone refers to the area within the aneurysm where the inflow stream collides with the aneurysm wall, resulting in a change in its direction and/or dispersal. Inflow concentration occurs when inflow streams or jets penetrate relatively deeply into the aneurysm sac, exhibiting a thin or narrow profile in the primary flow direction. For the figures shown, the blood flow of aneurysm model was simulated using streamline, moreover, a cross-sectional cutting plane at the neck and aneurysmal sac are also depicted. The WSS distribution is displayed as a color contour map over the aneurysm.

## Results

**Fig. 4:**
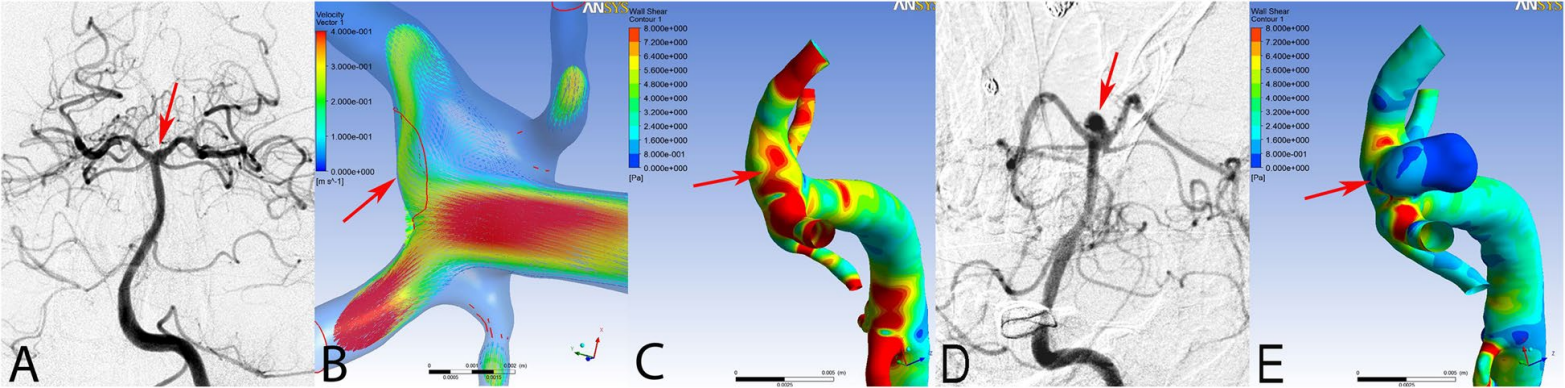
Hemodynamic characteristics of aneurysmal initiation and growth. In the region of aneurysmal initiation (**A**–**C**), high flow impingement impacted the basilar tip vessel wall continuously (arrow in **B**). The WSS distribution showed a local maximum at the apex of the initiation process of the aneurysmal formation (arrow in **C**). However, after aneurysmal growth (**D**, **E**), the region of aneurysmal growth showed a low WSS magnitude at peak systole (arrow in **E**)

Three aneurysms were analyzed using a hemodynamic simulation. Based on the hemodynamic simulation, the flow velocity of the aneurysm and of the aneurysmal neck showed significant reductions in the stabilized aneurysm, and the continuing impact of blood flow at the neck was shown in the recanalized aneurysm. (Fig 3) The distribution of WSS magnitudes on the aneurysmal sac showed that the ruptured aneurysms had a lower WSS than the unruptured one (Fig 2). During the initiation process of aneurysm formation, observations revealed high flow impingement and a concentrated distribution of high WSS on a small segment of the arterial wall (Fig 4B, C). After aneurysmal growth, the WSS distribution decreased with the progression of the new sac.

### Hemodynamic results for the ruptured aneurysm

For the first SAH, the AcomA aneurysm was considered to be a ruptured aneurysm (Fig 1D, E). The value of WSS distribution on ruptured AcomA aneurysm (2.66 Pa) was lower relative to unruptured right PcomA aneurysm (4.22 Pa). (Fig 1F, arrows; Fig. 2). For the second SAH (Fig 1G, H), the basilar tip aneurysm also showed a lower WSS (0.85 Pa) at the aneurysmal sac (Fig 1I, arrow; Fig. 2). Moreover, the value of the aneurysmal WSS distribution was quantified in Figure 2, the value of WSS on ruptured basilar tip aneurysm was lowest, followed by ruptured AcomA aneurysm and then unruptured PcomA aneurysm.

### Hemodynamic results for the stable and recanalized aneurysms

On the 18-month follow-up angiograph, the PcomA aneurysm was stable and the AcomA aneurysm showed recanalization at the aneurysmal neck. Compared with pretreatment simulations, the flow concentration of the AcomA aneurysm was still evident, while the flow of the PcomA aneurysm was modified well following embolization with coils alone (Fig 3D, H, arrows). High-flow concentration was still noticeable in the middle area of the aneurysmal neck and dome when examining the velocity magnitude on the cutting plane (Fig 3J, arrow), and a high flow concentration region was found on the neck plane after embolization (Fig 3L, arrow). Remarkably, the area exhibiting high-flow velocity corresponded precisely with the recanalization site as verified by follow up angiography. In contrast, the flow concentration of the stable PcomA aneurysm was modified completely and the flow concentration region on the neck plane was reduced after embolization compared with the pre-embolization model (Fig 3M–P).

### Hemodynamic results for aneurysmal initiation and growth

During the first treatment, there was no aneurysm observed in the tip of the basilar artery. However, a newly-initiated, irregular basilar artery tip aneurysm with a daughter bleb was observed at the 18-month follow-up (Fig 4). In the hemodynamic simulations of the period before aneurysmal initiation, the WSS distribution showed a local maximum at the apex of the initiation process of the aneurysmal formation. (Fig 4B) High flow impingement impacted the basilar tip vessel wall continuously. However, after aneurysmal growth, the aneurysmal dome showed a low WSS distribution at peak systole (Fig 4C, E).

## Discussion

In this study, we create a unique model of multiple aneurysms that exhibited aneurysmal initiation, rupture, and recanalization. After hemodynamic simulation, we discover that high flow concentration area on the neck plane perisisted in recanalized aneurysm after coil embolization, and low WSS was the risk hemodynamic factor for aneurysmal rupture. For the hemodynamics of aneurysmal initiation, a high flow impingement and a high WSS distribution before aneurysmal initiation were found, while the WSS distribution and flow velocity were decreased in the new sac after aneurysmal growth.

### Hemodynamics of the aneurysmal rupture

In previous studies, ruptured aneurysms have lower WSS values than those of unruptured aneurysms [9–12]. Localized low WSS values caused by stasis of blood flow or intra-aneurysmal thrombosis are believed to indicate local inflammatory responses within the arterial wall, with chronic exposure potentially causing ongoing wall degeneration and thinning, and finally rupture. Similar results were also found in some studies with multiple aneurysms. However, only hemodynamics of aneurysm rupture was investigated in these studies [9, 13–17]. In our study, we present a unique model with multiple aneurysms, which initiation, growth, rupture, and recurrence occurred during the follow-up time. We found that the results for the ruptured AcomA and basilar tip aneurysms were similar to our previous results. However, attempts to define the hemodynamic characteristics of the aneurysmal rupture have been controversial. Cebral et al. and Castro et al. [18, 19] reported that ruptured aneurysms were more likely to have a larger maximum WSS; however, Xiang et al. [20] found that intracranial aneurysm rupture could be predicted by a low WSS and a high oscillatory shear index. These opposing views may be explained by the study of Kono et al. [21], which reported that simulations of the ruptured aneurysm may be inaccurate when the aneurysm changes shape after rupture and that changes in aneurysm shape after rupture should be considered in CFD research. As a result, the hemodynamic results for aneurysms following rupture may differ from aneurysms before rupture.

### Hemodynamics of aneurysmal recanalization

Previous studies have identified high-flow velocity at the residual neck or within the neck region as a contributory factor that poses a risk for recanalization. [4, 22, 23]. In the case presented here, recanalization occurred where the velocity was higher at the neck plane after embolization with coils alone, which supports our previous results. The following underlying mechanisms may explain such results: (1) one possible explanation is that the high residual velocity at the neck plane could induce compaction of the coils, ultimately resulting in aneurysm recurrence; (2) another possibility is that the continued transmission of blood concentration may hamper the local flow stasis process and prevent thrombosis formation in the aneurysm [24]; finally, the higher residual velocity may interfere with neointima formation at the aneurysmal orifice, which is thought to be essential to ensure complete healing of embolized aneurysms [25, 26]. The size of the aneurysm might influence hemodynamic results as Jou et al. reported and they found no WSS-derived hemodynamic variables related to aneurysm size [27].

### Hemodynamics of aneurysmal initiation and growth

The precise hemodynamic mechanism capable of initiating an aneurysm remains incompletely understood. With regard to aneurysmal initiation, Sforza et al. [28] reported that the WSS is an important hemodynamic factor that causes an imbalance in the homeostatic condition of the endothelial layer, and leads to aneurysmal initiation and progression. Several studies have shown that aneurysmal initiation is related to flow acceleration, which causes a high WSS. High WSS is associated with different gene expression by endothelial cells in the affected region, and causes vascular remodeling [29, 30]. There is agreement among studies on the role of the hemodynamic factors in aneurysmal formation, but the exact mechanism of aneurysmal growth is still unclear [2, 31]. Low WSS and high WSS theories have tried to elucidate the underlying mechanism for this. The low WSS theory has been supported by several studies [2, 32, 33]. According to the low WSS theory, endothelial cells exposed to low WSS have a higher probability of aneurysmal progression. Moreover, low WSS increases permeability, enabling inflammatory cell infiltration of the endothelial layer induced by nitrous oxide [2, 34]. On the other hand, results for the high WSS theory have shown that high WSS may cause endothelial damage, proinflammatory changes and vascular remodeling, supporting aneurysmal growth [31]. This process leads to an imbalance between high aneurysmal WSS and high blood flow impingement, inducing aneurysmal formation and growth [35]. However, Sugiyama et al. [36] performed a hemodynamic study of tandem aneurysms to investigate the hemodynamic differences between the two growing aneurysms. They found that growing regions of an aneurysm could be exposed to either high WSS at the inflow zone or low WSS in the aneurysm sac. In our study, high flow impingement impacted the basilar tip vessel wall, while a high WSS distribution was observed at the apex in the region of aneurysmal initiation and growth. However, after aneurysmal growth, the high WSS region of the aneurysm changed to a low WSS distribution, which might have caused the aneurysmal rupture.

### Limitations

 Firstly, it's important to note that our study was limited to a single case, highlighting the need for larger sample sizes to comprehensively investigate the hemodynamic characteristics associated with aneurysmal initiation, rupture, and recanalization. Secondly, our CFD simulations utilized assumptions common in many studies, including rigid walls, laminar flow, and Newtonian blood properties. However, these assumptions may not fully capture the complexity of real-world hemodynamics, especially since flow conditions were not patient-specific. Therefore, there is a potential for these simplifications to impact the accuracy of our hemodynamic findings. Validation in larger cohorts is necessary to confirm our results. Lastly, hemodynamic characteristics may be one factor that influences aneurysmal initiation, rupture, and recurrence, but other pathologic factors may also influence the outcome.

## Conclusions

We present a unique model of multiple aneurysms that exhibited aneurysmal initiation, rupture, and recanalization. Hemodynamic simulations revealed that the aneurysmal initiation was accompanied by a high flow impingement and a high WSS distribution, and that after aneurysmal growth, the WSS distribution and flow velocity were decreased in the new sac. In addition, the aneurysmal rupture was associated with a markedly low WSS. The reduction of the high flow concentration region on the neck plane after coil embolization may be an important factor for protecting recanalization of the aneurysm.

## Data Availability

Not applicable.

## Abbreviations

AcomA: Anterior communicating artery
PcomA: Posterior communicating artery
CFD: Computational fluid dynamics
SAH: Subarachnoid hemorrhage
mRS: Modified Rankin scale
DSA: Digital subtraction angiography
3D: Three-dimensional
CT: Computed tomography
WSS: Wall shear stress
